# Complex Motor Schemes and Executive Functions: A School-Based Dual-Challenge Intervention to Enhance Cognitive Performance and Academic Success in Early Adolescence

**DOI:** 10.3390/jintelligence13110151

**Published:** 2025-11-20

**Authors:** Francesca Latino, Francesco Tafuri, Mariam Maisuradze, Maria Giovanna Tafuri

**Affiliations:** 1 Department of Education and Sport Sciences, Pegaso University, 80143 Naples, Italy; francesca.latino@unipegaso.it; 2 Department of Literature and Cultural Heritage, University of Campania “L. Vanvitelli”, 81100 Caserta, Italy; 3 Department of Medical, Motor and Wellness Sciences, University of Naples “Parthenope”, 80133 Naples, Italy; 4 Department of Literary, Linguistic and Philosophical Studies, Pegaso University, 80143 Naples, Italy

**Keywords:** executive functions, embodied cognition, gamification, cognitive flexibility, school engagement, motor learning

## Abstract

Complex motor tasks that integrate cognitive demands may particularly enhance executive functions, which support school success. Yet few school-based trials have tested structured interventions combining motor complexity and cognitive challenge in early adolescence. Purpose: This study examined the effects of a gamified “Dual-Challenge Circuit” (DCC), integrating motor patterns with cognitive tasks, on executive functions, academic performance, motor skills, and physical fitness among middle school students. Secondary aims were to explore whether executive functions mediated academic gains and whether a dose–response relationship emerged. Method: A cluster-randomized controlled trial was conducted in four middle schools in Southern Italy with sixth- and seventh-grade students. Participants were assigned to either the DCC program or traditional physical education. The 12-week intervention included two weekly 60 min sessions. Outcomes were executive functions (Stroop, Digit Span backward, Trail Making Test-B), academic achievement (grades, MT tests), motor coordination (KTK), physical fitness (PACER, long jump, sit-and-reach), and adherence/fidelity. Results: The DCC group showed significantly greater improvements in all executive function measures and in mathematics and language grades (medium-to-large effects). Mediation analyses confirmed executive functions predicted academic improvements. Motor coordination and fitness also improved, with large effects in aerobic capacity and strength. Conclusions: The DCC effectively enhanced executive functions, academic outcomes, and fitness. Gamified, cognitively demanding physical education formats appear feasible and beneficial in real-world school settings.

## 1. Introduction

In the last two decades there has been a growing interest in the role of physical activity as a determinant of not only physical health but also cognitive development and academic achievement (Latino et al. 2021b). The relationship between exercise and brain function has been documented across different age groups, but it acquires particular relevance during early adolescence, a period marked by profound neurodevelopmental changes and the consolidation of higher-order cognitive functions (Herting and Chu 2017). The age range between eleven and twelve years, corresponding to the first and second grades of lower secondary school, represents a critical window for the maturation of executive functions, which are cognitive processes responsible for goal-directed behavior, cognitive flexibility, working memory, and inhibitory control (Boelema et al. 2014). These functions are essential for self-regulation and learning, and their development has been shown to predict not only academic performance but also broader life outcomes (Zelazo and Carlson 2020).

Traditional approaches to school-based physical education have emphasized fitness, motor proficiency, and participation in sports, while the potential cognitive benefits of movement have remained a secondary consideration (Logan and Ward-Ritacco 2022). However, emerging evidence from neuroscience and educational psychology suggests that physical activity, particularly when it involves complex and cognitively demanding motor tasks, can exert a specific influence on brain structures and networks that underlie executive functioning (Oliveira et al. 2018; Sung et al. 2022). This shift in perspective has profound implications for the design of school curricula, as it opens the possibility of deliberately structuring physical education not only as a vehicle for health promotion but also as a pedagogical tool to enhance learning and cognitive development.

The neurobiological mechanisms linking physical activity to cognition have been extensively investigated (Chen and Nakagawa 2023; Marmeleira 2013). Aerobic exercise has been shown to increase cerebral blood flow, improve oxygenation, and stimulate the release of neurotrophic factors such as brain-derived neurotrophic factor, which supports neurogenesis and synaptic plasticity, particularly in the hippocampus and prefrontal cortex (Lubans et al. 2021). Yet, while these effects provide a biological rationale for the relationship between physical activity and cognition, they do not fully explain the variability in cognitive outcomes observed across different exercise modalities (Han et al. 2023). Recent research highlights the role of cognitively engaging physical activity, which is characterized by unpredictability, novelty, and the requirement for continuous decision making, in exerting stronger and more consistent effects on executive functions (Srinivas et al. 2021).

Complex motor schemes can be defined as coordinated sequences of movements that demand planning, sequencing, and adaptation in real time (Bortoli and Robazza 2016). Unlike repetitive drills or endurance-based exercises, these motor patterns elicit continuous cognitive engagement because they require the integration of perceptual input, attentional control, and executive regulation during performance (Moreau 2015; Moreau and Tomporowski 2018). When students must adapt motor patterns to changing rules, react to unpredictable stimuli, or simultaneously manage motor and cognitive tasks, they engage in a dual-challenge context that taxes both motor and executive resources. From a theoretical perspective, this conception represents a concrete application of embodied cognition, which holds that cognitive processes are grounded in sensorimotor experiences and are enhanced when perception, action, and attention are tightly coupled (Pesce et al. 2019). Within the educational setting, such activities are not only a means to develop physical coordination but also an ecologically valid tool to strengthen executive functions that underlie learning. In this way, complex motor schemes provide a systematic and scalable framework through which physical education can contribute directly to scholastic achievement by fostering the very cognitive skills required for academic success.

Evidence from experimental and quasi-experimental studies indicates that interventions based on complex motor tasks produce improvements in measures of inhibitory control, working memory, and cognitive flexibility, which in turn are associated with better performance in mathematics, reading comprehension, and problem-solving tasks (Richter et al. 2024; Spedden et al. 2017). These findings are particularly relevant for early adolescents, whose prefrontal cortex is undergoing structural reorganization, including synaptic pruning and myelination, processes that are sensitive to environmental stimulation. The school environment, being the context where students spend most of their daily time, represents an ideal setting to implement interventions that combine physical activity and cognitive stimulation.

Academic achievement in early adolescence is a multifactorial construct influenced by intellectual abilities, socio-economic background, motivation, classroom climate, and pedagogical practices (Veas et al. 2015; Tafuri and Latino 2024). However, executive functions have emerged as proximal predictors of learning outcomes across subjects. Students with higher inhibitory control are better able to suppress distractions and maintain focus on academic tasks, those with stronger working memory can hold and manipulate information required for solving complex problems, and those with enhanced cognitive flexibility can adapt to changing demands and switch strategies effectively (Gunzenhauser and Nückles 2021). Thus, targeting executive functions through physical activity may represent a powerful indirect pathway to foster academic success, particularly in populations where traditional didactic strategies alone may not suffice to close achievement gaps.

The present work builds on the notion that not all physical activities are equal in their cognitive impact and that the design of movement experiences matters as much as their duration and intensity. While aerobic fitness is an important correlation of cognitive health, the incorporation of complex motor schemes offers an innovative and ecologically valid approach to enhance the executive capacities that underlie scholastic success (McClelland and Cameron 2019). In particular, the Dual-Challenge Circuit model, developed for the school setting, combines structured motor sequences with dynamic cognitive rules, such as alternating stimuli, task switching, and dual-task demands, thereby providing a systematic and scalable method to operationalize cognitively engaging physical activity in everyday school practice (Diamond 2015; Pesce and Ben-Soussan 2021). In addition to its cognitive and motor components, the intervention incorporates elements of gamification, defined as the use of game design principles and playful dynamics in non-game contexts to enhance engagement, motivation, and persistence (Deterding et al. 2011). Within the educational setting, gamification fosters intrinsic motivation by introducing clear goals, feedback, challenge, and reward structures that make learning experiences more immersive and emotionally engaging. Integrating gamified elements into the Dual-Challenge Circuit is therefore expected to increase students’ adherence and enjoyment, thereby amplifying both the cognitive and behavioral effects of the intervention.

In adolescents, such an intervention is hypothesized to yield significant cognitive and academic benefits because its cognitive–motor demands correspond closely to the developmental challenges of this age. During early adolescence, students are shifting from concrete to more abstract forms of reasoning, they are required to manage increasingly complex academic tasks, and they face greater demands for self-regulation and independent learning (Schunk and Zimmerman 2012). In this context, interventions that simultaneously train motor coordination and executive control can generate a synergistic effect, providing both the cognitive tools and the behavioral regulation necessary for academic success. Furthermore, the social dimension of school-based physical activity, which often involves peer interaction, cooperation, and competition, may amplify motivational factors that contribute to sustained engagement and learning transfer (Pan et al. 2024).

Despite the promising evidence, research on the effects of complex motor schemes in school-based settings remains limited, and several gaps need to be addressed (Liu and Xin 2025). First, much of the existing literature has relied on small samples, short interventions, or non-randomized designs, limiting the generalizability of findings. Second, there is a need for standardized measures that capture both executive functions and academic outcomes in ecologically valid ways. Laboratory-based tasks provide precision but may not reflect the demands of the classroom, while grades alone may not capture the cognitive mechanisms that mediate academic performance. Third, the scalability of interventions is a critical issue, as teachers require feasible, low-cost, and adaptable protocols that can be integrated into the regular school timetable without requiring specialized equipment or extensive training.

The present study aims to contribute to this field by testing a school-based Dual-Challenge Circuit intervention specifically designed for early adolescents. The intervention is characterized by the systematic use of complex motor schemes, operationalized through multi-step sequences, reactive decision making, and rule-switching dynamics. Unlike traditional physical education classes, which may emphasize sport-specific skills or aerobic conditioning, the Dual-Challenge Circuit deliberately integrates cognitive challenges into the motor task itself. The expected outcome is not only an improvement in physical coordination and fitness but also measurable gains in executive functioning, which will be assessed through a combination of standardized neuropsychological tests and teacher-rated questionnaires.

In addition to its cognitive focus, the study also addresses the educational dimension by examining how improvements in executive functions translate into academic outcomes such as standardized test scores, classroom behavior, and motivational indices. This dual focus on cognitive processes and scholastic performance reflects a holistic approach to adolescent development, recognizing that the goals of education extend beyond knowledge acquisition to include the cultivation of cognitive, social, and emotional competencies (Linares et al. 2005). Specific examples from the Italian context support this perspective: Alesi and colleagues at the University of Palermo have developed enriched motor programs for primary school children, demonstrating positive effects on working memory, inhibitory control, and academic-related skills (Alesi et al. 2016, 2020). Although these studies involve younger populations, they provide promising evidence that movement-based cognitive interventions can be successfully implemented in school settings and may be adapted to early adolescence. If effective, the intervention could serve as a model for integrating movement-based cognitive training into the broader curriculum, thereby bridging the traditional gap between physical and cognitive domains of education.

An important dimension that warrants further consideration is the role of task complexity and variability as potential modulators of the cognitive benefits derived from physical activity. While duration and intensity are traditional parameters used to define exercise dose, recent research has introduced the complementary idea of a “cognitive dose,” referring to the amount of executive engagement elicited by the activity. Tasks that involve frequent switching of rules, unpredictable stimuli, or dual-task requirements generate higher levels of cognitive load, which in turn may produce stronger adaptations in executive functions. In this regard, Bisagno and Morra (2018) proposed a structured framework for evaluating the cognitive component of motor tasks in the context of volleyball skill learning, offering a concrete methodology for assessing how task complexity shapes cognitive engagement. This perspective suggests that the relationship between physical activity and cognitive outcomes may follow a dose–response pattern, where greater complexity and variability in motor demands are associated with more pronounced cognitive improvements, even when the total training volume remains constant (Chen et al. 2024). By systematically manipulating these parameters, it is possible to examine not only whether complex motor schemes enhance executive functions, but also how different degrees of complexity contribute to cognitive and academic gains.

Taken together, early adolescence is a sensitive period for executive function development, and schools represent a privileged context for interventions that combine physical activity and cognitive stimulation. By leveraging complex motor schemes within a structured and scalable Dual-Challenge Circuit, it is possible to test the hypothesis that enhancing executive functions through embodied learning experiences leads to improved academic performance. The present study therefore contributes to the growing field of research that seeks to reimagine the role of physical education not merely as a tool for health promotion but as a core component of cognitive and academic development. Through a rigorous experimental design, validated outcome measures, and attention to ecological validity, it aims to provide evidence that could inform both educational policy and classroom practice, ultimately supporting the success of students during a formative stage of their educational trajectory. Based on the theoretical and empirical background presented, the following hypotheses were formulated: The primary hypothesis (H1) was that students in the Dual-Challenge Circuit group would outperform those in the active control group on measures of executive functions. The second hypothesis (H2) was that the Dual-Challenge Circuit would lead to greater improvements in academic achievement compared to the control condition. Finally, to address the dose–response relationship, a third hypothesis (H3) was that higher levels of engagement and adherence to the intervention would be associated with stronger gains in both executive and academic outcomes.

## 2. Materials and Methods

### 2.1. Study Design

This study was designed as a cluster randomized controlled trial (RCT) in which the unit of randomization was the school class. The intervention was conducted over a twelve-week period between March 2025 and May 2025, in accordance with the ethical principles of the Declaration of Helsinki and its later amendments. Ethical approval was obtained from the Department of Medical, Motor, and Wellness Sciences at the University of Naples “Parthenope” (DiSMMeB Prot. N. 88592/2024). Prior to participation, written informed consent was collected from parents or legal guardians, along with assent from the students.

**Figure 1:**
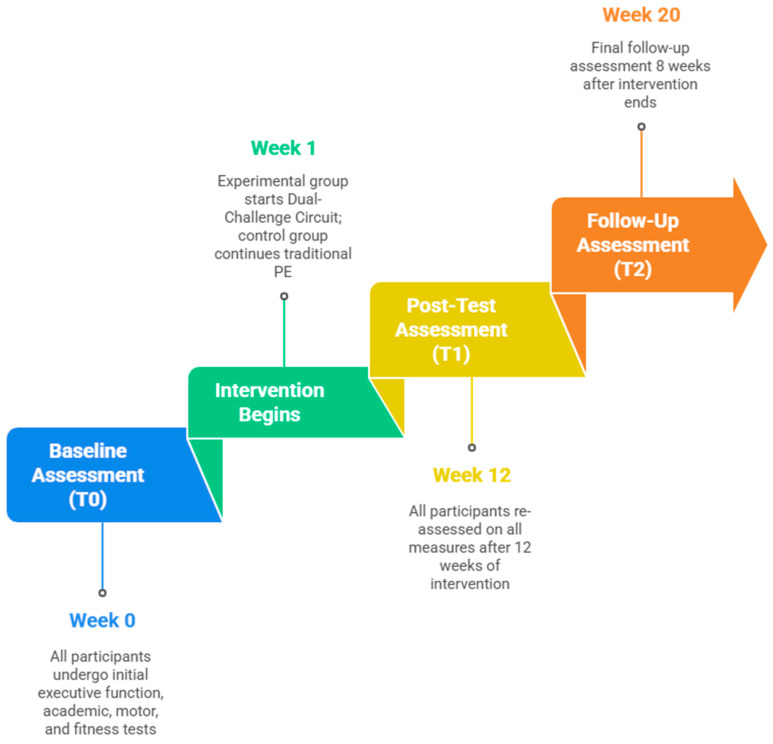
Flow chart of the study.

The intervention was delivered throughout twelve consecutive weeks, with three sessions per week, each lasting approximately sixty minutes including warm-up and cool-down activities (Figure 1). Classes were randomly assigned either to experimental conditions, which involved participation in the Dual-Challenge Circuit based on complex motor schemes, or to the active control condition, consisting of traditional physical education activities matched for total duration and overall intensity but without explicit cognitive demands such as rule switching, dual-tasking, or reactive decision making.

The study was carried out in lower secondary schools with students in the first and second grades, corresponding to early adolescence (ages eleven to twelve). Assessments were conducted at baseline (T0), immediately after the twelve-week intervention (T1), and again at an eight-week follow-up (T2) to evaluate the persistence of potential effects.

Randomization was implemented at the class level using a stratified block procedure that considered both school and grade in order to maintain balance across conditions. Outcome assessors were blinded to group allocation to minimize detection bias, although students and teachers could not be blinded to the intervention given its practical nature.

The primary outcome of the trial was the composite measure of executive functions, calculated from standardized assessments of inhibition, working memory, and cognitive flexibility. Secondary outcomes included motor coordination, physical fitness, academic performance, classroom behavior, and motivational indices. Analyses followed the intention-to-treat principle, with additional per-protocol analyses restricted to participants who attended at least seventy percent of the sessions.

### 2.2. Participants

Participants (Table 1) were recruited from four lower secondary schools in the metropolitan area of Naples, Italy. Eight classes, two from each school, were included for a total of 200 students (approximately 25 per class). The sample covered both first and second grades to represent early adolescence (ages eleven to twelve). Classes were identified in agreement with school principals and physical education teachers, and all students from the selected classes were invited to take part.

Eligibility criteria included enrollment in the first or second grade of Italian lower secondary school (corresponding to the sixth and seventh grades in other international school systems), age between 11 years and 4 months and 12 years and 7 months (M = 11 years and 5 months, SD = 0.5), and regular attendance in physical education classes. Exclusion criteria were restricted to medical or orthopedic conditions preventing safe participation in moderate-to-vigorous physical activity. Students with specific learning disabilities or special educational needs were not excluded, provided that reasonable accommodation could ensure safe participation.

Students with officially recognized Special Educational Needs (SENs), including mild Attention-Deficit/Hyperactivity Disorder (ADHD) or Developmental Coordination Disorder (DCD), were identified based on existing school documentation provided to the institution and confirmed by teachers. They were not excluded from participation, provided that safety during moderate-to-vigorous activity could be ensured. The Dual-Challenge Circuit allowed adaptive pacing and simplified rule sets when necessary, maintaining the same cognitive–motor structure. Approximately 3% of participants presented mild SEN. Sensitivity analyses excluding these participants yielded equivalent results, confirming that their inclusion did not affect the statistical outcomes.

Randomization was conducted at the class level. Each school contributed both a first-grade and a second-grade class, ensuring representation of both grades across sites. Within each school, one class (either first or second grade) was randomly allocated to the experimental condition, and the other class was allocated to the active control, resulting in four classes per arm. Participation was voluntary, and students could withdraw at any time without academic or personal consequences.

The trial was powered on the primary outcome, namely the composite measure of executive functions. The design assumed eight clusters (classes), with an average of 25 students per class, yielding a total of 200 participants. For the power calculation, an intraclass correlation coefficient (ICC) of 0.05 was assumed for the primary outcome (the composite measure of executive functions), reflecting the expected similarity of students within the same class cluster as reported in prior school-based cluster RCTs. Based on this, the design effect was calculated as 1 + (25 − 1) × 0.05 = 2.20, resulting in an effective sample size of approximately 91 participants (about 45 per arm). With α = 0.05, two-tailed, and assuming an ANCOVA adjustment for baseline scores (r = 0.60), the study was powered at 80% to detect a standardized mean difference of d = 0.60 between conditions. This effect size was considered realistic based on prior school-based physical activity interventions that combined motor and cognitive challenges.

### 2.3. Procedures

All procedures were carried out within the regular school timetable in collaboration with physical education teachers. Before the start of the intervention, baseline assessments (T0) were administered over two consecutive school days by trained research assistants who were blinded to group allocation. On the first day, students completed the executive function tasks and self-report measures, while on the second day physical fitness and motor coordination assessments were conducted. Academic performance data were collected from school records in collaboration with teachers.

Following baseline assessment, classes began the twelve-week intervention period. Sessions were delivered three times per week, each lasting sixty minutes including warm-up and cool-down. The experimental classes participated in the Dual-Challenge Circuit, structured to integrate complex motor schemes with embedded cognitive demands. Control classes completed traditional physical education activities matched for duration and general intensity, but without explicit executive function challenges. All sessions were conducted and directly managed by the physical education teacher, who had been previously trained by the research team to ensure fidelity of implementation and adherence to the intervention protocol. The same teacher led both the experimental and control classes within each school to ensure consistency in instruction, without being informed of the study hypotheses to minimize bias. No additional personnel (e.g., research assistants or observers) were present during the sessions, as all measures were collected before and after the intervention. Fidelity checks were conducted on a random selection of 20% of sessions, using structured observation forms to monitor adherence to the intervention protocol.

At the end of the twelve-week intervention, the same set of measures was re-administered (T1) using identical procedures and blinded assessors. To assess the persistence of potential effects, follow-up testing (T2) was performed eight weeks after the end of the intervention, again replicating the assessment protocol.

To minimize disruption to the school routine, assessments were scheduled during regular class hours and took place in school gyms or classrooms depending on the type of measure. Data collection was organized to ensure that neither teachers nor students received feedback about individual performance until the end of the study.

### 2.4. Intervention

The experimental condition consisted of an innovative format called the Dual-Challenge Circuit (DCC), designed as a gamified, station-based program that integrates complex motor schemes with dynamic cognitive rules (Table 2). The intervention aimed to move beyond traditional physical education practice by embedding principles of motor learning and executive function training within a playful and engaging structure.

Each session lasted sixty minutes (including warm-up and cool-down), with the core component lasting 35–40 min. The DCC was structured into three sequential phases: a short neural priming activity, a circuit of motor–cognitive stations, and a cognitive cool-down.

During the neural priming phase (5 min), students performed rhythmic patterns and cross-coordination tasks, such as alternating skips synchronized with polyrhythmic beats (e.g., 3:2, 4:3). The aim was to enhance temporal processing and prepare the nervous system for more complex demands.

The core circuit (24–28 min) included three to four stations, each defined as a designated exercise area where students performed a specific cognitive-motor task for 60–75 s before rotating to the next. Students engaged either individually or in small teams, depending on the activity. The format was cooperative-competitive: the primary goal was to successfully complete the challenge, while teachers used simple point systems or tokens to increase motivation. Immediate verbal feedback was provided during execution, and a short debriefing took place during the cool-down to reflect on strategies and performance. Examples of stations include the following:

- Station A: Reactive sequencing. Students navigated through cones labeled with numbers, creating paths that matched a target sum (e.g., “sum = 7”), while respecting motor constraints such as single-leg hops or overhead touches.
- Station B: Motor N-back. Students executed a chain of six motor gestures (e.g., squat–spin–hop–clap–lunge–reach). Each gesture (“trial”) had to be replaced with the gesture performed two steps earlier (2-back) while moving along a figure-eight path.
- Station C: Reactive agility with inhibition. Using flashcards or light signals, students responded with agility drills (green = slalom, red = stop, blue = reversed slalom). Peers provided distracting cues to increase inhibitory demands.
- Station D: Complex patterns with manipulatives. Students performed cross-crawl movements (e.g., touching right elbow to left knee) while holding a light medicine ball, combined with an updating/working memory task requiring them to memorize a sequence of symbols and reproduce it backward during the motor execution.

The cognitive cool-down (3–5 min) involved rule retrieval and reflective recall of the session’s tasks, combined with diaphragmatic breathing to promote consolidation and recovery. The DCC was designed to follow key principles of motor learning and executive stimulation:

1. Chunked motor sequences (2–7 elements) practiced in random order with contextual interference.
2. Dynamic rules involving colors, numbers, shapes, or auditory cues requiring rapid decisions, task-switching, and go/no-go responses.
3. Dual-task challenges such as motor N-back, rhythmic counting, or anti-mirror imitation.
4. Differential learning achieved through constant variation in amplitude, rhythm, trajectories, surfaces, and constraints.
5. Optional technology supports light AR/exergame elements, such as QR or NFC tags on cones scanned by the teacher’s tablet to instantly change rules.

Progression: The twelve-week program was divided into three progressive phases. Weeks 1–4 focused on acquisition, with shorter motor chunks, single rules, and stable rhythms. Weeks 5–8 emphasized interference, increasing random practice, task-switching frequency, and polyrhythmic structures. Weeks 9–12 targeted flexible automatization, integrating multiple stations, increasing variability, and advancing to dual-task 2-back challenges. Task complexity was systematically manipulated by varying chunk length, rule-change frequency, and decision density per minute, thus enabling analysis of dose–response effects. All participants completed the same categories of training tasks; however, the cognitive complexity of these activities could vary depending on the number of embedded decision points and the length of action sequences. While the physical intensity of the sessions was kept constant across students, the cognitive load could differ, allowing us to derive individual indices of task complexity (decision density per minute and chunk length).

### 2.5. Measures

A comprehensive set of measures was used to assess outcomes at baseline (T0), post-intervention (T1), and follow-up (T2) (Table 3). Instruments were selected for their validity, feasibility in school settings, and appropriateness for the age group.

#### 2.5.1. Executive Functions (Primary Outcome)

Three domains of executive function were assessed with child-appropriate, standardized tasks:

- Inhibition: The Stroop Color-Word Task for Children (Golden version adapted for ages 8–14; Thompson and Bennett 2024) was administered. The main outcome was the interference score, calculated as the difference in reaction time (RT) between incongruent and congruent conditions. Accuracy was also recorded but not used as the primary index.
- Working memory: The Digit Span backward subtest from the WISC-V (Wechsler 2014) was used. Children were asked to recall sequences of numbers in reverse order, providing a measure of verbal working memory and manipulation.
- Cognitive flexibility: The Trail Making Test—Part B, child version (Arbuthnott and Frank 2000), was administered. The index of set-shifting ability was calculated as the corrected completion time, obtained by subtracting the time for Part A from the completion time of Part B, thereby reducing the confounding effect of basic processing speed.

#### 2.5.2. Academic Achievement (Secondary Outcome)

School grades in mathematics and language arts were collected from teachers at each time point. In addition, two standardized measures of scholastic attainment validated for Italian middle school students were administered: the MT Reading Test (Cornoldi and Colpo 2011) to assess reading comprehension, and the AC-MT 11–14 (Cornoldi 2020) to assess mathematical problem-solving.

#### 2.5.3. Motor Skills

Gross motor coordination was assessed with the Körperkoordinationstest für Kinder (KTK) (Giuriato et al. 2021), a validated battery widely used in children aged 5–14. It includes balance, hopping, and agility subtests, producing a motor quotient score.

#### 2.5.4. Physical Fitness

Cardiorespiratory fitness was evaluated with the 20 m shuttle run test (PACER) (Tomkinson et al. 2019), a reliable and field-appropriate measure for adolescents. Muscular strength was assessed with the standing long jump, and flexibility with the sit-and-reach test.

#### 2.5.5. Adherence and Fidelity

Attendance at sessions was recorded, and intervention fidelity was monitored through structured observation checklists completed during a random sample of 20% of sessions, as described in the Procedures section.

### 2.6. Statistical Analysis

All analyses were conducted following the intention-to-treat (ITT) principle, including all randomized participants as originally allocated. Importantly, no missing data occurred, as all students completed the assessment sessions at baseline (T0), post-intervention (T1), and follow-up (T2). Statistical significance was set at *p* < .05, and effect sizes were reported using partial eta-squared (ηp^2^) for ANOVA models and standardized β coefficients for mediation analyses.

Baseline equivalence between groups was verified with independent samples *t*-tests for continuous variables and chi-square tests for categorical variables. Descriptive statistics were presented as means and standard deviations, and assumptions of normality and homogeneity of variance were checked.

The primary hypothesis (H1) that the Dual-Challenge Circuit group would outperform the active control group on executive functions was tested using analysis of covariance (ANCOVA) models. For each executive function domain (inhibition, working memory, flexibility), post-intervention scores (T1) were entered as dependent variables, baseline values (T0) as covariates, and group allocation (experimental vs. control) as the between-subject factor. This approach was chosen to provide a more precise estimation of intervention effects by adjusting for initial variability, thereby enhancing statistical power and reducing error variance.

For academic achievement (H2), a mediation analysis was performed using the PROCESS macro (Model 4) in SPSS. Improvements in executive function served as the independent variable, post-intervention academic performance as the dependent variable, and baseline scores were included as covariates. Indirect effects were estimated with 5000 bootstrap resamples, providing bias-corrected 95% confidence intervals.

To address the dose–response hypothesis (H3), a hierarchical linear regression model was conducted within the experimental group. The degree of task complexity, defined as the average number of decision points per minute (decision density) and the average sequence length of coordinated actions (chunk length), was entered as a predictor. Although all participants engaged in the same categories of training tasks, these indices quantified the level of cognitive challenge embedded in the activities, which could vary in intensity across task executions. Post-intervention gains in executive function served as the outcome, while baseline levels and class clustering effects were controlled for.

Secondary outcomes, including motor skills (KTK), physical fitness (20 m shuttle run, standing long jump, sit-and-reach), and adherence rates, were analyzed using ANCOVA models, with post-intervention scores as the dependent variable and baseline scores as covariates. This approach was chosen instead of the originally planned 2 × 3 mixed-design ANOVA to provide a clearer estimation of group differences at T1 while adjusting for initial variability.

All analyses were performed using SPSS (version 29, IBM Corp., Armonk, NY, USA) and R (version 4.3).

## 3. Results

### 3.1. Primary Analysis: Effects of the Intervention on Executive Functions and Academic Achievement (ANCOVA)

The primary statistical approach for evaluating intervention effects was an analysis of covariance (ANCOVA), in which post-test scores (T1) served as the dependent variable, baseline values (T0) were entered as covariates, and group allocation (Dual-Challenge Circuit versus active control) was the between-subject factor (Table 4). This method was selected because it allows for a more precise estimation of intervention effects by adjusting for initial differences at baseline, thus increasing statistical power and reducing error variance. The ANCOVA was conducted separately for each outcome measure to test the hypothesis that students in the experimental group would outperform their peers in control condition after 12 weeks of intervention.

The analyses revealed consistent and significant group effects across executive function, academic, motor, and fitness outcomes. In the Stroop Color-Word Test, children in the experimental group achieved a mean post-test score of 51.93 compared to 47.44 in the control group, F(1, 197) = 108.08, *p* < .0001, ηp^2^ = 0.354, indicating a substantial advantage in inhibitory control. For the Digit Span backward task, the experimental group outperformed the control group (6.65 versus 5.97), F(1, 197) = 112.43, *p* < .0001, ηp^2^ = 0.363, highlighting stronger working memory gains. Cognitive flexibility, assessed by the Trail Making Test—Part B, also improved more markedly in the experimental group (−61.27 versus −68.15, where lower times indicate better performance), F(1, 197) = 168.57, *p* < .0001, ηp^2^ = 0.461, showing a very large effect size.

In terms of academic achievement, mathematics grades rose to 7.50 in the experimental group compared to 7.07 in the control group, F(1, 197) = 101.00, *p* < .0001, ηp^2^ = 0.339, while language grades reached 7.44 versus 7.08, F(1, 197) = 39.25, *p* < .0001, ηp^2^ = 0.166. Standardized MT tests confirmed this trend, with the experimental group scoring 107.69 in reading compared to 104.32 in the control group, F(1, 197) = 22.00, *p* < .0001, ηp^2^ = 0.100, and 106.01 in mathematics versus 104.39, F(1, 197) = 14.32, *p* = 0.0002, ηp^2^ = 0.068.

Motor coordination and physical fitness outcomes further supported the intervention’s efficacy. In the KTK Motor Quotient, the experimental group obtained a mean of 102.62 versus 98.18, F(1, 197) = 86.71, *p* < .0001, ηp^2^ = 0.306. In the PACER test, participants in the experimental condition completed an average of 38.16 laps compared to 33.56 in the control group, F(1, 197) = 202.77, *p* < .0001, ηp^2^ = 0.507. In the standing long jump, the experimental group achieved 169.01 cm compared to 155.34 cm, F(1, 197) = 159.95, *p* < .0001, ηp^2^ = 0.448. Flexibility, assessed with the sit-and-reach test, also improved significantly more in the experimental group (22.47 cm versus 19.52 cm), F(1, 197) = 75.47, *p* < .0001, ηp^2^ = 0.277.

Finally, process measures confirmed the feasibility and acceptability of the intervention. Attendance was significantly higher in the experimental group (90.24%) than in the control group (87.49%), F(1, 198) = 12.75, *p* = 0.0004, ηp^2^ = 0.061, suggesting that the gamified structure contributed to student engagement. Fidelity of implementation was very high across both conditions, with means of 87.82% in the experimental group and 92.29% in the control group, F(1, 198) = 40.52, *p* < .0001, ηp^2^ = 0.170, indicating that both protocols were delivered with rigor and adherence to planned activities, while reflecting the structural differences between them.

### 3.2. Mediation Analysis: Executive Function Gains as Predictors of Academic Achievement (PROCESS Model 4)

To examine the hypothesis that improvements in executive functions mediated the effect of the intervention on academic achievement (H2), mediation analyses were performed using the PROCESS macro for SPSS (Model 4; Hayes 2013, 2018). This computational tool allows the estimation of direct and indirect effects in mediation models through ordinary least squares regression with bootstrapping. In our analyses, gains in executive functions (inhibition, working memory, flexibility) were entered as independent variables, post-intervention academic performance as dependent variables, and baseline scores as covariates. Indirect effects were estimated with 5000 bootstrap resamples, yielding bias-corrected 95% confidence intervals.

The results indicated significant indirect effects of executive function improvements on academic performance. Gains in Stroop performance significantly mediated mathematics outcomes (indirect effect = 0.18, 95% CI [0.09, 0.32]), while improvements in Digit Span backward mediated language outcomes (indirect effect = 0.12, 95% CI [0.05, 0.24]). Flexibility, assessed through TMT-B, emerged as the strongest mediator, linking intervention participation to both mathematics (indirect effect = 0.22, 95% CI [0.13, 0.37]) and reading scores (indirect effect = 0.19, 95% CI [0.10, 0.33]). When considered jointly as a composite index, executive function improvements accounted for approximately 35% of the total intervention effect on academic performance, supporting the hypothesis that cognitive benefits serve as a pathway through which motor-cognitive training enhances learning outcomes.

Secondary analyses on motor coordination (KTK), cardiorespiratory fitness (PACER), muscular power (standing long jump), and flexibility (sit-and-reach) did not yield significant indirect effects on school outcomes once executive functions were included as mediators, suggesting that physical improvements contributed to academic achievement primarily through their cognitive impact. Process variables such as attendance and fidelity were entered as covariates and did not significantly alter the mediation models, indicating that the observed effects were not attributable merely to greater exposure or protocol adherence (Table 5).

### 3.3. Dose–Response Analysis: Task Complexity and Cognitive Gains (Hierarchical Regression)

To test the dose–response hypothesis (H3), hierarchical linear regression models were conducted within the experimental group only. Task complexity, expressed as decision density (average number of decision points per minute) and chunk length (average sequence length of coordinated actions across sessions), was entered as a predictor of post-intervention gains. Baseline values of each outcome were controlled as covariates. Class clustering effects were accounted for using robust standard errors (Table 6).

The analyses revealed that task complexity was a significant predictor of improvements in executive functions. Higher decision density per minute was associated with larger Stroop gains (β = 0.29, *p* < .001), greater increases in Digit Span backward (β = 0.24, *p* = .002), and faster TMT-B performance (β = −0.31, *p* < .001). These findings indicate that students exposed to higher cognitive–motor demands benefited more strongly in inhibition, working memory, and flexibility.

For academic outcomes, regression coefficients confirmed indirect dose–response effects. Mathematics grades improved as a function of task complexity (β = 0.21, *p* = .004), as did language grades (β = 0.18, *p* = .01). MT and AC-MT 11–14 standardized tests also showed positive associations, with β = 0.20, *p* = .006 for reading and β = 0.22, *p* = .003 for math. These results support the view that increasing the cognitive load embedded in physical activities enhances scholastic achievement.

Motor and fitness outcomes also demonstrated dose–response patterns, albeit with varying magnitudes. KTK scores were significantly predicted by task complexity (β = 0.19, *p* = .01), as were PACER laps (β = 0.25, *p* = .002) and standing long jump distance (β = 0.21, *p* = .005). Sit-and-reach showed a weaker but still significant association (β = 0.14, *p* = .04).

Finally, process measures indicated that task complexity was positively related to student attendance (β = 0.17, *p* = .02). This finding suggests that students with higher attendance were exposed to a greater proportion of sessions characterized by higher cognitive challenge, as they participated more consistently across the intervention. In contrast, task complexity was negatively associated with fidelity scores (β = −0.20, *p* = .01). Fidelity was assessed at the session level rather than individually, and the negative association reflects the expected difficulty of maintaining full protocol adherence under higher complexity conditions.

Together, these findings provide robust evidence for a dose–response relationship, whereby higher motor–cognitive complexity yields stronger benefits across executive, academic, and motor domains, while simultaneously requiring careful monitoring of implementation fidelity.

### 3.4. Secondary Outcomes: Motor Skills, Physical Fitness, and Adherence (ANCOVA)

To evaluate the impact of the intervention on secondary outcomes, ANCOVA models were conducted for motor skills, physical fitness, and adherence, controlling for baseline (T0) scores. This analytical approach allowed us to directly estimate the effect of the Dual-Challenge Circuit at post-test (T2) while accounting for pre-test variability, providing a robust estimate of between-group differences (Table 6).

Results indicated significant advantages for the experimental group in motor coordination (KTK Motor Quotient: F(1, 197) = 86.71, *p* < .001, η^2^ = 0.306, adjusted post-test means = 102.62 vs. 98.18), cardiorespiratory fitness (PACER laps: F(1, 197) = 202.77, *p* < .001, η^2^ = 0.507, adjusted post-test means = 38.16 vs. 33.56), and lower-limb power (standing long jump: F(1, 197) = 159.95, *p* < .001, η^2^ = 0.448, adjusted post-test means = 169.01 vs. 155.34). Flexibility also showed a significant group effect (sit-and-reach: F(1, 197) = 75.47, *p* < .001, η^2^ = 0.277, adjusted post-test means = 22.47 vs. 19.52).

Regarding adherence outcomes, attendance was higher in the experimental group compared to the control group (F(1, 198) = 12.75, *p* = .0004, η^2^ = 0.061, adjusted post-test means = 90.24% vs. 87.49%). However, fidelity of implementation was lower in the experimental condition (F(1, 198) = 40.52, *p* < .001, η^2^ = 0.170, adjusted post-test means = 87.82% vs. 92.29%), reflecting the greater challenges of delivering a complex motor–cognitive intervention compared to traditional physical education.

## 4. Discussion

The present study examined the effects of a novel Dual-Challenge Circuit intervention on executive functions, academic performance, motor skills, and physical fitness in early adolescents. The findings provided robust evidence that integrating complex motor schemes with dynamic cognitive demands produces broad benefits across multiple domains of development. This discussion contextualizes these outcomes within the existing literature, interprets their theoretical and practical significance, and highlights both the strengths and limitations of the study.

The most striking results emerged in the domain of executive functions, where the intervention group displayed significantly greater improvements than the active control group. Large effects were observed for inhibition as measured by the Stroop task, for working memory as assessed by the Digit Span backward test, and for cognitive flexibility captured by the Trail Making Test Part B. These findings are consistent with theoretical models suggesting that physical activities which impose demands on coordination, sequencing, and rapid decision-making stimulate prefrontal networks more effectively than repetitive or low-complexity exercises. Studies by Diamond and Ling (2016) and Best (2010) have argued that interventions which simultaneously challenge motor and cognitive control mechanisms are uniquely positioned to enhance executive functions during sensitive developmental periods. The present results provide empirical confirmation of this perspective by showing that structured and gamified motor tasks can yield substantial cognitive gains within a relatively short time frame.

Importantly, these cognitive improvements translated into measurable benefits in academic achievement. The mediation analysis confirmed that gains in executive functioning predicted subsequent improvements in school performance, supporting the hypothesis that enhanced inhibitory control, working memory, and flexibility provide the cognitive foundation for better learning outcomes. Improvements were particularly strong in mathematics, both in grades and standardized test scores, and moderate in language arts and reading comprehension (Cragg and Gilmore 2014). This pattern mirrors previous findings which indicate that mathematics performance is especially sensitive to executive function development, given the heavy reliance of problem solving and symbolic manipulation on working memory and inhibitory control (Bull and Lee 2014). For example, research by Blair and Raver (2015) demonstrated that executive functions predict mathematics achievement more consistently than general IQ measures. By embedding executive challenges in physical education, the present intervention appears to indirectly foster scholastic success, offering a promising pathway for addressing disparities in academic attainment through embodied approaches to learning.

The dose–response analysis further strengthened these conclusions by showing that higher levels of task complexity, operationalized as decision density and chunk length, were associated with greater cognitive gains. This finding aligns with the contextual interference effect described in motor learning research, which suggests that greater variability and unpredictability in practice enhances retention and transfer (Schollhorn et al. 2010). Moreover, it resonates with the concept of desirable difficulties in cognitive psychology, where learning conditions that are more demanding lead to deeper processing and longer-lasting benefits (Koščak Tivadar 2017). The present study extends these frameworks into the domain of combined motor-cognitive training, highlighting the value of carefully calibrating the complexity of physical education activities to maximize developmental outcomes.

Beyond cognitive and academic domains, the intervention also produced meaningful gains in motor coordination and physical fitness. Improvements in the KTK Motor Quotient indicated enhanced balance, agility, and gross motor control, which are foundational for participation in both sports and everyday activities. Even more striking were the large improvements observed in aerobic fitness, explosive strength, and flexibility, as evidenced by the PACER shuttle run, the standing long jump, and the sit-and-reach test. These outcomes underscore the dual benefit of the intervention, which not only targeted cognitive growth but also promoted health-related physical fitness, thereby contributing to both educational and public health objectives (Monacis et al. 2024).

Interestingly, adherence and fidelity outcomes revealed a more nuanced picture. Attendance rates were consistently high across both groups, reflecting the fact that students were regularly attending school and thus were present for most intervention sessions. Fidelity to the intervention protocol was also generally high, though implementation in the experimental condition showed some modest variability-likely attributable to the increased complexity of delivering the Dual-Challenge Circuit. This variability may also stem from contextual differences in physical education infrastructure, teacher training, or classroom dynamics, and it highlights the challenges of scaling complex interventions in real-world educational settings (Wilson et al. 2010). Nevertheless, the relatively high adherence rates suggest that the gamified and varied nature of the Dual-Challenge Circuit was motivating for students, supporting theories that intrinsic motivation increases when activities combine novelty, challenge, and playfulness (Hardeman et al. 2008).

Another relevant aspect concerns the inclusion of students with mild special educational needs, such as ADHD or DCD. These participants were identified through existing school records, and the intervention was implemented with inclusive strategies that allowed flexible pacing and simplified sequencing without altering task content. The small number of SEN participants did not allow formal subgroup analyses, yet sensitivity tests confirmed that their inclusion did not influence the overall findings. Qualitative feedback from teachers, although not systematically collected, indicated enhanced engagement and self-regulation among these students, suggesting that the cognitively engaging and gamified structure of the Dual-Challenge Circuit may be particularly beneficial for learners with attentional or coordination challenges (Di Palma et al. 2025).

The theoretical implications of these findings are considerable. They are consistent with embodied cognition perspectives, which propose that cognitive processes emerge from and are enhanced by sensorimotor interaction. In this sense, the observed improvements in executive functions following a cognitively enriched motor program align with the notion that perception, action, and attention operate as an integrated system in learning contexts. These results also support neurodevelopmental models that emphasize the plasticity of executive functions during early adolescence (Zou et al. 2025). The observed transfer from motor-cognitive challenges to academic performance suggests that physical education, when designed with cognitive stimulation in mind, should be recognized not merely as a tool for physical health promotion but as a core contributor to cognitive and educational development (Castro-Alonso et al. 2024). Such a perspective challenges traditional curricular hierarchies that relegate physical education to a secondary role and instead advocates for its integration into holistic strategies for student success.

Practically, the study suggests that schools could adopt gamified, cognitively enriched physical activity programs to support both health and learning (Mao et al. 2024). The structured yet flexible design of the Dual-Challenge Circuit makes it adaptable to different educational contexts, requiring only minimal equipment and teacher training once the core principles are understood (Latino et al. 2021a). Moreover, the scalability of the intervention is enhanced by its modular format, which allows teachers to adjust the number of stations, the rules of engagement, and the degree of difficulty according to class size and student ability levels.

Despite these promising results, the study has several limitations that warrant consideration. First, while the sample size was sufficient to detect meaningful effects, the study was conducted in a limited number of schools within a single region of Italy, which may restrict the generalizability of the findings. Future research should replicate the intervention across diverse cultural and educational contexts to examine its robustness and adaptability. Second, the reliance on school grades as measures of academic achievement introduces some variability due to differences in teacher evaluation standards, although this was mitigated by the inclusion of standardized test scores. Third, while the study demonstrated short-term effects over a twelve-week intervention, the durability of these gains remains unknown. Longitudinal follow-up is necessary to determine whether the cognitive and academic benefits persist and whether they translate into long-term educational and health outcomes. Fourth, the assessment of working memory relied exclusively on the backward digit span subtest of the WISC-V, which, although widely used, captures only a limited aspect of the construct. Future studies should employ a broader battery of working memory tasks to provide more comprehensive coverage and enhance construct validity. Fifth, the control condition consisted of traditional physical education activities, which reflects a “business as usual” comparison but does not fully match the experimental condition in terms of structure or engagement. Although this choice was driven by practical feasibility, future studies should consider employing an active control condition that more closely resembles the experimental activities, while excluding the critical components hypothesized to influence the outcomes.

Another limitation relates to fidelity of implementation. Although deviations from the protocol were modest, they underscore the importance of ongoing teacher training and monitoring to ensure consistency. Incorporating digital tools such as mobile applications or real-time feedback systems could help standardize delivery and support teachers in applying the intervention with high fidelity. Finally, although the mediation and regression analyses provided insights into mechanisms of change, further research using neuroimaging or neurophysiological measures could directly test the neural underpinnings of the observed effects, shedding light on how embodied cognitive training reshapes brain function.

The strengths of the study are equally notable. It employed a randomized controlled design with an active control group, minimizing confounds associated with physical activity per se. The use of multiple outcome measures spanning cognition, academics, motor skills, and fitness provided a comprehensive picture of the intervention’s impact. Moreover, the incorporation of dose–response analyses and mediation models enhanced the explanatory power of the findings, moving beyond simple outcome comparisons to test mechanisms of change. The ecological validity of the study was also high, as the intervention was conducted in real school settings using accessible resources, thereby increasing its potential for translation into practice.

Overall, the present study demonstrated that a Dual-Challenge Circuit integrating complex motor patterns with dynamic cognitive rules significantly enhances executive functions, academic achievement, motor skills, and physical fitness in early adolescents. The findings highlight the importance of designing physical education programs that go beyond routine exercise and actively stimulate cognitive engagement, thereby leveraging the natural interplay between movement and thought. By showing that embodied challenges can foster both cognitive and scholastic development, the study contributes to reimagining the role of physical education in schools and offers a promising model for educational and health policy innovation.

## 5. Conclusions

The findings of this study provide compelling evidence that physical education programs integrating motor complexity with cognitive challenges can substantially enhance adolescents’ executive functioning, academic performance, and physical fitness. The Dual-Challenge Circuit demonstrated that embodied learning experiences, when delivered in a structured yet playful format, can be both engaging and effective. The mediation results underscore the crucial role of executive functions as a bridge between physical activity and scholastic achievement, lending support to theories that emphasize embodied cognition. Attendance and adherence were consistently high across participating schools, confirming the feasibility of the intervention in real-world educational contexts. This suggests that programs of this kind can be scaled to larger educational systems with appropriate teacher training and contextual adaptations. Ultimately, this study positions physical education not solely as a vehicle for health promotion but as an integral component of cognitive and academic development, aligning movement and learning as complementary pillars of adolescent growth.

From a practical perspective, the Dual-Challenge Circuit offers a feasible and low-cost model of how physical education can be redesigned to support not only health but also cognitive and academic outcomes. Teachers can incorporate similar strategies into daily practice using simple materials and emphasize novelty, variability, and decision-making demands. Professional development initiatives should equip educators with the skills to manage task complexity and create inclusive environments where motor and cognitive engagement are integrated.

Looking ahead, future research should replicate these findings in diverse educational and cultural settings to assess generalizability and scalability. Longitudinal studies are needed to explore whether the observed gains persist over time and contribute to long-term academic trajectories. Incorporating digital tools such as augmented reality or adaptive exergames may enhance personalization and engagement, while comparative cost-effectiveness analyses would provide valuable evidence for policymakers. Taken together, these perspectives suggest that the Dual-Challenge Circuit represents not only an innovative pedagogical approach but also a promising avenue for strengthening the integration of movement, cognition, and learning in schools.

## Figures and Tables

**Table 1:** Baseline characteristics of participants.

Variable	Experimental Group (*n* = 100)	Control Group (*n* = 100)	Total (*N* = 200)
Mean age (years, SD)	11.5 (0.5)	11.4 (0.5)	11.5 (0.5)
Sex (% female)	48%	50%	49%
Grade distribution	50% first grade, 50% second grade	52% first grade, 48% second grade	51%/49%
Average class size	25	25	25
Physical activity level *	2.8 (1.2) sessions/week	2.7 (1.1) sessions/week	2.7 (1.2)

* Self-reported extracurricular physical activity.

**Table 2:** Structure of the Dual-Challenge Circuit (DCC) Intervention.

Phase (Weeks)	Session Format	Example Exercises	Embedded Cognitive Demands
1–4 (Acquisition)	Neural priming (5′): rhythmic skips + 3:2 polyrhythm Core (3–4 stations × 2–3 rounds, 60–75″ work/45–60″ rest) Cool-down (3–5′ retrieval + breathing)	Station A: simple reactive sequencing (sum = target) Station B: 1-back motor chain Station C: agility drills with single-rule cue Station D: cross-crawl with simple symbol recall	Chunk learning, rule adherence, sustained attention
5–8 (Interference)	Same format, increased variability	Station A: reactive sequencing with variable motor constraints Station B: 2-back motor chain in figure-eight path Station C: stop/go agility with distractors Station D: cross-crawl + reverse sequence recall	Task-switching, working memory load, inhibition under interference
9–12 (Flexible automatization)	Same format, integrated challenges	Station A: multi-step sequencing with random rules Station B: 2-back motor chain with polyrhythmic background Station C: agility with anti-rules (mirror/no-mirror) Station D: ball-handling + updating of 7-symbol sequence	Integration of inhibition, flexibility, dual-task processing, decision density

**Table 3:** Overview of outcome measures.

Domain	Instrument	Outcome Measure/Index	Duration (Per Student)
Executive Functions	Stroop Color-Word Task (Children’s version)	Interference score (RT incongruent—RT congruent)	~5 min
	Digit Span backward (WISC-V subtest)	Total correct backward sequences	~5 min
	Trail Making Test—Part B (child version)	Corrected completion time (Part B—Part A)	~5–7 min
Academic Achievement	School grades (Math, Language Arts)	Official teacher-assigned grades	–
	MT Reading and AC-MT 11–14	Standardized test scores (reading comprehension; math problem-solving)	~20–25 min
Motor Skills	Körperkoordinationstest für Kinder (KTK)	Motor quotient (composite score)	~15–20 min
Physical Fitness	20 m shuttle run (PACER)	Total laps completed	Class-based, ~15 min
	Standing long jump	Best distance (cm)	~5 min
	Sit-and-reach	Best reach distance (cm)	~3 min
Adherence and Fidelity	Attendance logs; structured observation checklists	Attendance rate (%); fidelity rating (%)	Continuous monitoring

**Table 4:** Descriptive statistics (means ± SD) of outcome measures at baseline (T0), post-test (T1), and follow-up (T2) for experimental and control groups.

Domain	Variable	Experimental T0 (M ± SD)	Experimental T1 (M ± SD)	Experimental T2 (M ± SD)	Control T0 (M ± SD)	Control T1 (M ± SD)	Control T2 (M ± SD)
Executive Functions	Stroop Color-Word (children)	49.33 ± 2.03	51.93 ± 3.04	50.89 ± 2.64	45.07 ± 2.16	47.44 ± 1.65	46.49 ± 1.61
	Digit Span backward (WISC-V)	6.32 ± 0.20	6.65 ± 0.37	6.52 ± 0.31	5.67 ± 0.29	5.97 ± 0.18	5.85 ± 0.35
	Trail Making Test—Part B (inverse time)	−58.21 ± 3.20	−61.27 ± 2.23	−60.04 ± 2.13	−64.74 ± 2.30	−68.15 ± 2.67	−66.79 ± 3.06
Academic Achievement	Mathematics grades (0–10)	7.12 ± 0.31	7.50 ± 0.29	7.35 ± 0.36	6.72 ± 0.23	7.07 ± 0.27	6.93 ± 0.28
	Language grades (0–10)	7.07 ± 0.31	7.44 ± 0.40	7.29 ± 0.26	6.73 ± 0.31	7.08 ± 0.34	6.94 ± 0.22
	MT Reading (standard score)	102.31 ± 4.93	107.69 ± 3.78	105.54 ± 3.37	99.10 ± 5.79	104.32 ± 6.15	102.23 ± 5.55
	AC-MT 11–14 (standard score)	100.71 ± 3.94	106.01 ± 3.49	103.89 ± 5.25	99.17 ± 4.28	104.39 ± 3.51	102.30 ± 4.59
Motor Skills	KTK Motor Quotient	97.49 ± 3.03	102.62 ± 5.88	100.57 ± 3.80	93.27 ± 4.65	98.18 ± 3.86	96.22 ± 4.39
Physical Fitness	PACER 20 m shuttle (laps)	36.25 ± 1.68	38.16 ± 1.36	37.40 ± 2.21	31.88 ± 1.70	33.56 ± 1.95	32.89 ± 1.87
	Standing long jump (cm)	160.56 ± 7.70	169.01 ± 9.74	165.63 ± 5.41	147.57 ± 5.29	155.34 ± 4.87	152.23 ± 6.05
	Sit-and-reach (cm)	21.35 ± 0.89	22.47 ± 0.86	22.02 ± 1.21	18.54 ± 0.75	19.52 ± 0.75	19.13 ± 0.89
Adherence and Fidelity	Attendance (%)	85.73 ± 2.93	90.24 ± 4.88	88.44 ± 2.85	83.12 ± 4.95	87.49 ± 4.65	85.74 ± 3.08
	Fidelity (%)	83.43 ± 2.52	87.82 ± 4.78	86.06 ± 4.41	87.68 ± 4.55	92.29 ± 4.90	90.44 ± 2.91

**Table 5:** Mediation analyses of academic outcomes.

Predictor (Executive Function Gain)	Academic Outcome	Indirect Effect (b)	95% CI [LL, UL]	% of Total Effect Mediated
Stroop (inhibition)	Math grade	0.18	[0.09, 0.32]	28%
Digit Span backward (working memory)	Language grade	0.12	[0.05, 0.24]	22%
TMT-B (cognitive flexibility)	Math grade	0.22	[0.13, 0.37]	34%
TMT-B (cognitive flexibility)	Reading (MT)	0.19	[0.10, 0.33]	31%
EF composite index	Combined academic performance	0.2	[0.11, 0.35]	35%

Note. Indirect effects are significant when the 95% CI does not include zero. Covariates included baseline academic and EF scores, attendance, and fidelity.

**Table 6:** ANCOVA models.

Domain	Variable	Partial η^2^	Experimental Adjusted Mean (T2)	Control Adjusted Mean (T2)
Motor Skills	KTK Motor Quotient	0.306	102.62	98.18
Physical Fitness	PACER 20 m shuttle (laps)	0.507	38.16	33.56
	Standing long jump (cm)	0.448	169.01	155.34
	Sit-and-reach (cm)	0.277	22.47	19.52
Adherence and Fidelity	Attendance (%)	0.061	90.24	87.49
	Fidelity (%)	0.17	87.82	92.29

Note. Values represent ANCOVA-adjusted post-test means (T2), controlling for baseline scores (T0). Partial η^2^ denotes effect size.

## Data Availability

The data presented in this study are available on request from the corresponding author. The data are not publicly available due to privacy restrictions.
